# Integrative Transcriptome, Genome and Quantitative Trait Loci Resources Identify Single Nucleotide Polymorphisms in Candidate Genes for Growth Traits in Turbot

**DOI:** 10.3390/ijms17020243

**Published:** 2016-02-17

**Authors:** Diego Robledo, Carlos Fernández, Miguel Hermida, Andrés Sciara, José Antonio Álvarez-Dios, Santiago Cabaleiro, Rubén Caamaño, Paulino Martínez, Carmen Bouza

**Affiliations:** 1 Departamento de Xenética, Facultade de Bioloxía (CIBUS), Universidade de Santiago de Compostela, Santiago de Compostela 15782, Spain; diego.robledo@usc.es; 2 Departamento de Xenética, Facultade de Veterinaria, Universidade de Santiago de Compostela, Lugo 27002, Spain; carlos.fernandez.lopez@usc.es (C.F.); miguel.hermida@usc.es (M.H.); paulino.martinez@usc.es (P.M.); 3 Instituto de Biología Molecular y Celular de Rosario (IBR-CONICET), Rosario S2002LRK, Argentina; asciara@ibr-conicet.gov.ar; 4 Departamento de Matemática Aplicada, Facultade de Matemáticas, Universidade de Santiago de Compostela, Santiago de Compostela 15782, Spain; joseantonio.alvarez.dios@usc.es; 5 Cluster de Acuicultura de Galicia (Punta do Couso), Aguiño-Ribeira 15695, Spain; cabaleiro@cetga.org (S.C.); ruben@cetga.org (R.C.)

**Keywords:** *Scophthalmus maximus*, turbot, growth, RNA-seq, candidate genes, SNP, integrative genomics

## Abstract

Growth traits represent a main goal in aquaculture breeding programs and may be related to adaptive variation in wild fisheries. Integrating quantitative trait loci (QTL) mapping and next generation sequencing can greatly help to identify variation in candidate genes, which can result in marker-assisted selection and better genetic structure information. Turbot is a commercially important flatfish in Europe and China, with available genomic information on QTLs and genome mapping. Muscle and liver RNA-seq from 18 individuals was carried out to obtain gene sequences and markers functionally related to growth, resulting in a total of 20,447 genes and 85,344 single nucleotide polymorphisms (SNPs). Many growth-related genes and SNPs were identified and placed in the turbot genome and genetic map to explore their co-localization with growth-QTL markers. Forty-five SNPs on growth-related genes were selected based on QTL co-localization and relevant function for growth traits. Forty-three SNPs were technically feasible and validated in a wild Atlantic population, where 91% were polymorphic. The integration of functional and structural genomic resources in turbot provides a practical approach for QTL mining in this species. Validated SNPs represent a useful set of growth-related gene markers for future association, functional and population studies in this flatfish species.

## 1. Introduction

Genetic dissection of polygenic traits is key for animal breeding and evolutionary genetics. Genomic studies offer the possibility of unraveling a huge amount of genetic polymorphisms useful for searching selection signatures in populations and for association studies with productive traits, such as growth or disease resistance, which could eventually be applied in marker-assisted selection (MAS) programs. The application of MAS has enabled increasing productivity in livestock previously subjected to traditional phenotype-based selection [1]. A more effective selection can be performed the closer the marker is to the DNA element responsible for phenotypic differences, or even better, if the marker itself causes that difference. Markers within genes physiologically related to the trait of interest may potentially allow for a more straightforward selection [2]. For example, an intronic single nucleotide polymorphism (SNP) in the chicken growth hormone gene was associated with a 16.9% increase in body weight [3], while microsatellite variation in the growth hormone receptor promoter in cattle could account for 6.9% production improvement (Hale *et al.*, 2000) [4]. Moreover, the identification of gene-associated markers has been applied for detecting adaptive variation and genetic structure in wild and farmed populations of commercially exploited species, which is particularly of interest in order to define conservation and management units in marine fisheries [5,6].

These strategies require information on the target genes and also the discovery of suitable markers, a difficult task in non-model species with limited genomic resources. Methodologies using genomic information from related species allowed us to identify SNP markers associated with growth and reproduction in fish [7,8]. However, new generation sequencing has greatly facilitated marker identification in non-model species. In this regard, transcriptome sequencing using RNA-seq technology allows SNP identification at an affordable cost [9,10]. SNPs are abundant markers along the genomes (e.g., ~1 SNP/100 bp in fish [11]), suitable for assisted selection on a wide range of genomic scales relying on the linkage disequilibrium patterns. In addition, SNP variation may have functional meaning, involving not only non-synonymous amino acid substitutions but also affecting regulatory elements (transcription factor and microRNA binding sites, or splicing recognition sites) [12].

Growth is one of the most important traits in animal breeding and a target in most genetic breeding programs, also in aquaculture species [13]. While most terrestrial livestock such as cattle, pigs or chickens have been strongly selected for commercial traits, only around 10% of aquaculture species have undergone breeding programs [14]. Genomic technologies have increased our knowledge on the molecular basis of growth traits in fish [15]. Different genomic studies have been carried out to detect quantitative trait loci (QTL) for growth traits in finfish and identified candidate genes to be used in marker-assisted selection programs [16,17,18,19,20,21]. Nevertheless, the genetic effects and regulatory pathways underlying the growth rate are still poorly understood in teleosts as compared to mammals [22,23].

Turbot is a marine flatfish of commercial importance in Europe and China [24,25], whose culture in aquaculture facilities is a mature industry. The genomic resources available include a genetic map [26,27] and a recently assembled genome sequence [28]. Previous studies in turbot found several QTLs and associated markers for growth traits, major goals for selective breeding programs in this species [29,30]. In addition, genome scanning has been used for detecting loci involved in adaptive variation of turbot populations, some of them presumptively related to growth performance [31,32]. The integrative connection among these resources may have implications for fisheries management and genetic improvement in this species.

The main aim of this study was the detection and validation of SNP markers in turbot located in candidate genes involved in growth and associated with growth-related QTLs in this species. We used Illumina RNA-seq to obtain muscle and liver transcriptomes from control and fasted specimens enriched in growth regulation pathways, and used this information to identify growth-related genes and associated SNP markers. Integrating (positioning) our new transcriptome data with previous genetic map markers in the draft turbot genome sequence enabled us to locate the candidate transcripts and compare their positions with growth QTL markers reported in the turbot genetic map. SNPs located in 45 genes were selected based on their co-localization with QTLs and functional relevance for growth. These SNPs were validated in a turbot population and will be useful to perform association analysis with phenotypes for growth traits both in farmed broodstock and natural populations for fisheries management.

## 2. Results and Discussion

### 2.1. RNA-Seq and SNP Calling

A total of 19,326,140 muscle and 17,466,901 liver reads were obtained from Illumina sequencing, which enriched the transcriptomic background for turbot, useful for functional and comparative genomics in flatfish and teleosts. After trimming, 89.6% of the reads remained and were aligned against the draft turbot genome [28]. Muscle and liver transcriptomes were successfully assembled from the aligned reads, identifying 19,147 and 15,928 genes, respectively, and a total of 20,447 genes (Table 1). The figures found in our study are comparable to those obtained using genome-guided alignment for *Takifugu rubripes* swim bladder (17,249 genes) and gill (16,836 genes), which together accounted for 19,388 different genes [33]. Genomes are critical tools for obtaining realistic transcriptomes since, at present, *de novo* assemblers usually produce a higher number of genes with lower mean length and N50 (length for which the collection of all genes of that length or longer contains at least half of the sum of the length of all the genes). For example, *de novo* assembly of the ray-finned fish *Coilia nasus* liver transcriptome resulted in 65,129 genes with a mean size of 607 bp and N50 of 813 bp [34]. 

**Figure 1:**
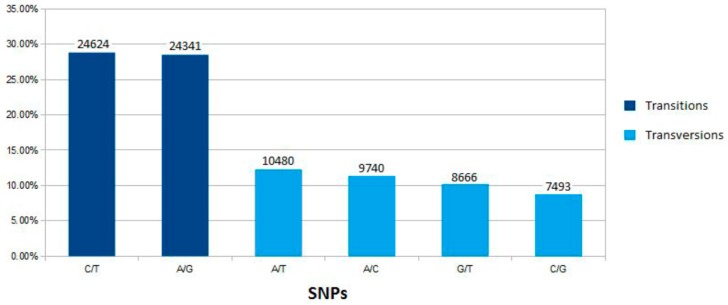
Single nucleotide polymorphisms (SNPs) detected in muscle and liver turbot RNA-seq. SNPs found by RNA-seq and aligned with turbot genomic sequences are separated according to their type.

Muscle and liver RNA-seq reads were used for SNP screening. A total of 93,558 polymorphisms were detected and 85,344 of them were SNPs (91.2%), similar to human estimates where SNPs account for 90% of genome variation [35]. The number of SNPs identified by next generation sequencing (NGS) technologies varies greatly among studies, probably due to different experimental designs (number of samples and tissues, sequencing depth, read length, genome availability, and SNP calling parameters, among others), but comparisons between transcriptome and genome data have been proven to improve experimental validation of SNPs [9,33,36,37]. As expected from previous studies in fish, most of the SNPs found in our work corresponded to A/G and C/T, each transition (ts) representing 28%, while transversion (tv) frequencies were below 13% (Figure 1). The ts/tv ratio of 1.346 is very similar to that previously reported in turbot based on 866 predicted SNPs obtained from two 454 Roche runs [38], and also consistent with the values found in common carp (*Cyprinus carpio*) (1.31) [39] and gilthead seabream (*Sparus aurata*) (1.37) [40]. Values were slightly higher in channel (1.56; *Ictalurus punctatus*) and blue (1.68; *Ictalurus furcatus*) catfish [37], but lower in other species, such as chum (*Oncorhynchus keta*) and sockeye (*Oncorhynchus nerka*) salmon (around 1) [41], close to the ratio estimated from intron regions in *C. carpio* (1.05) [39]. Still, these values are far from those in the human exome (3.2) [42] or in the whole nuclear genome (2.1) [43]. Besides the particular sampling scenario of each study, discrepancies may also be related to different selective pressures in the species under study [39].

Both muscle and liver transcriptomes were scanned for a large set of genes (~200) involved in growth regulation previously reported in fish [16,18,22,31,32]. We found 160 growth-related genes in muscle and 125 in liver, which account for a total of 174 different genes involved in growth processes. Furthermore, different isoforms were identified for some genes, totaling 325 different transcripts (1.86 isoforms per gene). Although in fish species the growth hormone (*gh*) has been described in liver [44], and the neuropeptide Y (*npy*) both in liver and muscle [45], we did not detect muscle or liver expression of these important growth-related hormones, nor did we find liver or muscle expression of prolactin (*prl*), consistent with the results in *Takifugu rubripes*, where *prl* was only found in the pituitary gland [46]. Hence, future growth-related studies should consider including brain as a target tissue.

**Figure 2:**
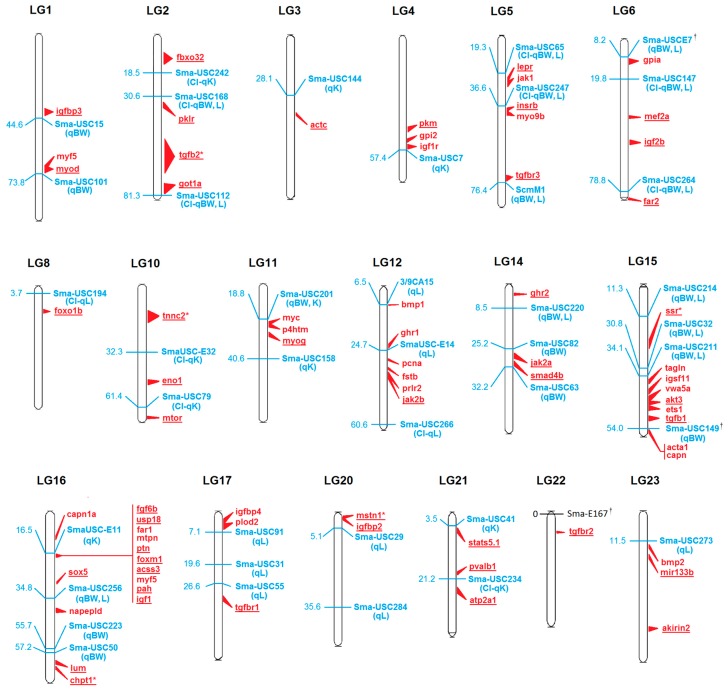
Predictive genome position of growth-related genes in the turbot genetic map. Estimated gene map positions are shown in red, underlining the 45 selected genes for SNP detection. (*) Map positions inferred by comparative mapping against model fish [26]. Reported growth-related markers [29,30,32] are shown in blue, either associated with growth traits (qBW, body weight; qL, length; qK, Fulton factor) or located within the confidence interval (CI) of growth QTLs. (†) Outlier markers proposed to be under selection in turbot [32].

All growth-related transcripts found in this study were located in the turbot genome and anchored to the genetic map using common markers [27,28]. Sixty-four genes were located in the vicinity of a previously reported growth QTL marker [29,30], within the same scaffold (Figure 2; Table S1, Supporting Information), and at an average distance of 1.9 Mb (~4 cM, assuming a relationship between genetic and physical distance of 0.5 Mb/cM [47]). The pooled population sample used for RNA sequencing in this study allowed the detection of SNPs for all these growth-related genes, which were selected for subsequent analyses. The number of SNPs per gene ranged from 1 to 25, with a mean of 7.81 ± 6.00.

We selected 45 out of 176 growth-related genes (those found in our transcriptome from an initial list of ~200) based on their relevance in growth-related processes in fish and vertebrates, their co-localization with growth QTL markers in turbot and the availability of technically suitable SNPs in the transcriptome (Table 2; Figure 2; Table S1, Supplementary Materials). According to Gene Ontology (GO) term classification, these genes were involved in different growth-related processes such as cell signaling, proliferation and growth (36%), energy metabolism (31%), muscle growth and development (26%) or cartilage and bone formation (7%) (Table S2, Supplementary Materials). We focused on the relationship between growth QTLs and candidate genes as a way to tackle the genetic basis underlying phenotypic effects, but we also focused on the gene relevance in growth-related processes [16,22] (Table 2). Previous QTL screening was performed with a limited amount of markers and families at early hatchery or at on-growing culture phases [29,30], so we would expect to find other genomic regions responsible for growth differences at different ages and under variable family or population genetic backgrounds. The selection included candidate genes associated with growth traits in different vertebrate and fish species, such as insulin growth factors 1 and 2 (*igf1* and *igf2*) and leptin receptor (*lpr*) [48,49,50], or myogenic regulatory factors such as myogenin (*myog*) associated with weight in pigs and chickens [51,52] and with indeterminate growth in fish [53]. Members of the transforming growth factor-beta (*tgfb*) pathway, which regulates cell growth and differentiation, were also included, since they were associated with growth and reproduction in vertebrates [54,55,56,57], such as, for example, myostatin 1 (*mstn1*). Null mutations in this negative regulator gene of muscle development in mammals lead to 30% increased growth in mice or to a “double muscle” phenotype in cattle [58,59]. SNP variation in the 3′ and 5′ untranslated regions (UTR) of myostatin was also associated with differences in growth traits in bighead carp (*Hypophthalmichthys nobilis*) [60] and Atlantic salmon (*Salmo salar*) [61], respectively. Other relevant genes selected were the parvalbumin 1 (*pvalb1*), where microsatellite variation at the 3′ UTR was associated with weight in Asian seabass (*Lates calcarifer*) [62] and growth hormone receptor 2 (*ghr2*), which has been associated with growth in cattle and chicken [4,63] and tilapia (*Oreochromis niloticus*, *O. aureus* and *O. mossambicus*) [23].

One SNP was selected for each gene among those available. Sixty percent of the 45 SNPs selected were located within the UTR (1:2 ratio in 5’ and 3’ UTR) and 31% within coding regions. Among the latter, five and nine SNPs were non-synonymous and synonymous mutations, respectively, whereas the others were located within introns of putative alternative transcript variants, as the alignment with orthologous genes from model fish suggested. These data could also be explained by the sequencing of immature transcripts, although intron retention seems to be a general phenomenon playing a role in the regulation of gene expression [64]. Non-synonymous mutations can directly alter the protein structure and function, since they cause substitutions in the amino acid sequence. Variation in the 5’ UTR can affect transcription factor binding sites, thus altering gene expression, while 3’ UTRs contain microRNA binding targets and can influence translation efficiency and mRNA stability [65]. Finally, some microRNA genes and consensus motifs required for correct splicing are found in introns [12,66]. Indeed, an intronic SNP in the growth hormone—Releasing hormone (*ghrh*) was associated with growth differences in Arctic charr (*Salvelinus alpinus*) [67].

### 2.2. SNP Variation

Sequenom assays were designed for the selected 45 SNPs into two multiplex reactions (Table S3, Supplementary Materials) for validation and genetic diversity estimation. Two SNPs were not technically feasible (*acss3*, *fbxo32*) and four were monomorphic in the tested population (*fgf6b*, *ghr2*, *got1a*, *myod*). Thus, a total of 43 SNPs were genotyped (95.6%) and 39 (86.7%) were finally polymorphic enough for diversity analysis in the Cantabrian population sample assayed (Table 3). Monomorphism at relevant growth-related loci in this wild population may be due to genetic differences between the Cantabric population and the samples used for RNA-seq SNP discovery, coming from a breeding strain of Atlantic origin. Accordingly, all feasible growth-related loci may be useful for further studies in other genetically divergent turbot populations of wild or farm origin [31,32]. Interestingly, some genic SNPs in this study were located close to outlier microsatellite and SNP markers proposed to be under divergent selection in turbot (e.g., SmaUSC-E7 at LG6; SmaUSC149 at LG15 or Sma-E167 at LG22; Figure 2) [32]. Gene-associated markers have become highly valuable tools for ascertaining origin assignment and detecting fine genetic structuration in fish [5,6]. Our panel of growth-related SNP markers will be a particularly useful tool to evaluate genetic differentiation patterns among wild Atlantic and Baltic populations with respect to farmed broodstock selected for growth in order to confirm changes in growth as an adaptation to differences in temperature and salinity conditions, which has implications for improving turbot aquaculture [32].

Among polymorphic SNPs, unbiased gene diversity (He) estimates ranged from 0.058 at *igf1*, *lum*, *pklr* and *tgfbr1* to 0.508 at *lepr* (mean: 0.304 ± 0.004). Minimum allele frequency (MAF) ranged from 0.029 (*igf1*, *lum*, *pklr* and *tgfbr1*) to 0.500 in *lepr* (mean: 0.221 ± 0.004). These values are in the range of those previously described in turbot [38,68] and other fish [39,69]. No departures from Hardy-Weinberg equilibrium (HW) were detected (α = 0.05; Table 3). F_IS_ values per locus and globally were not significantly different from zero at *p* < 0.05 and showed a mean value of −0.032 ± 0.0003 (Table 3), congruent with conformance to HW in the Atlantic turbot population when tested simultaneously for all loci (*p* = 1). Significant linkage disequilibrium (LD) at *p* < 0.05 was detected between 37 pairs of SNPs out of the total number of pairs of loci tested in the population under study (741 G-tests), which is close to the 5% expected by chance. Seven out of these 37 significant tests involved pairs of closely linked loci (19%), whereas the other cases involved markers in different linkage groups (LG) (mostly LG5, LG6, LG10 and LG16; Table S4). These cases can be related to type I errors, although epistatic interactions between loci located in different QTL regions cannot be discarded, as reported in fish for growth traits [17]. Only two significant LD tests were retained after Bonferroni correction (*p* = 0.00007), corresponding to two pairs of closely linked loci, *tnnc2*-*actc1* and *mstn1*-*igfbp2*, as predicted by the mapping of genomic scaffolds (Table S1, Supplementary Materials; Figure 2). Some closely linked genes derived from mapping predictions (Figure 2) were prioritized in this study to increase the probability of detecting informative SNPs within relevant QTL regions in different genetic backgrounds, particularly important for genes with low polymorphism. In summary, the set of polymorphic gene SNPs developed and validated in this study represents useful tools to be used in further population and family association studies in this species. The validation rate based on integrated RNA-seq and structural genomic resources in turbot (~90%) was much higher than in other recent RNA-seq strategies in fish: 54% (26/48) in *Takifugu rubripes* from swimbladder RNA-seq [33]; 56.9% in rainbow trout from muscle RNA-seq [9]. 

Although the number of SNPs and the true SNP ratio in RNA-seq may vary due to technical parameters, this technique allows us to obtain a much higher amount of SNPs than previous technologies. SNP calling based on 454 previous transcriptome runs in turbot only detected 866 SNPs, although the efficiency was rather good (79.3%) [38]. In cod, a different method involving PCR in 71 DNA fragments achieved a 37.1% success [7]. Overall, RNA-seq performed very well for obtaining gene-targeted SNPs at affordable costs, and it is particularly efficient if integrated with available structural genomic resources, as in our study. More importantly, we could focus on candidate genes within target QTL regions for traits of productive or evolutionary interest by integrating RNA-seq with physical and genetic mapping data for SNP identification.

### 2.3. Towards a High Density SNP Array in Turbot

Although the aim of this work was to generate an affordable panel of gene-linked SNPs co-localizing with QTL for growth association in a large amount of fish with different genetic backgrounds, the resources presented here can be further exploited at a larger genomic scale. The total number of SNPs generated in this study can be used for designing a more extensive SNP panel as a highly informative tool for studying the architecture of quantitative traits and for increasing the efficiency of selective breeding. Our aim would be the design of a large turbot SNP array based on this information and previous reports [38,70], and on the huge SNP amount obtained in an ongoing restriction site–associated DNA (RAD) sequencing project (>10 k SNPs; unpublished data), which could integrate growth-, reproduction- and immune-related gene-linked SNPs. Although large SNP panels are routinely employed in cattle or pigs for genomic selection, high density genotyping in a large number of individuals is still expensive for aquaculture. However, the availability of new cost-effective genotyping technologies has rendered the first high density genome-wide association studies (GWAS) in aquaculture species [71,72,73]. Still, high density SNP panels are scarce, and currently have been reported only in two oyster species, the Pacific oyster and the European flat oyster [74], and in several fish, Atlantic salmon [75], catfish [76], common carp [77] and rainbow trout [78]. More aquaculture high density SNP arrays will be available in incoming years and the SNPs reported here will surely contribute to this goal in turbot.

## 3. Experimental Section

### 3.1. Sampling for RNA Sequencing

Turbot juveniles from six unrelated families coming from a breeding strain of Atlantic origin were reared in tanks at water temperature of 18 °C at the facilities of CETGA (Aquaculture cluster of Galicia; Ribeira, Spain). Animals were divided in two groups, one fed daily and the other one subjected to nutritional stress by fasting. This study was performed on muscle and liver because these organs play a key role in the regulation of somatic growth in fish, the first one also representing the main edible part of fish [22,79]. In order to obtain the widest gene expression range and SNP variation among individuals as possible, muscle and liver samples were obtained from muscle and liver tissues of treated and control animals at 15 and 30 days after the start of the treatment: three fed as usual and six fasted fish per sampling time (total sample of 18 individuals). Individual samples were pooled per organ and embedded in RNA later for preservation (Qiagen, Valencia, CA, USA). Animals were treated according to the Directive 2010/63/UE of the European Parliament and of the Council of 22 September 2010 on the protection of animals used for experimentation and other scientific purposes. Experimental protocols were approved by the Institutional Animal Care and Use Committee of the University of Santiago de Compostela (Spain).

### 3.2. RNA Sequencing

RNA extraction was performed using the RNeasy mini kit (Qiagen) with DNase treatment following manufacturer’s instructions. RNA quality and quantity were evaluated in a Bioanalyzer 2100 (Agilent Technologies, Santa Clara, CA, USA) and in a NanoDrop^®^ ND-1000 spectrophotometer (NanoDrop Technologies Inc., Wilmington, NC, USA), respectively. Muscle and liver pooled samples were barcoded and sequenced on an Illumina HiSeq 2000 (100 bp paired-end) using standard protocols. Sequencing output quality was assessed using FastQC v0.11.2 [80]. Quality filtering and removal of residual adaptor sequences was conducted on read pairs using Trimmomatic v0.32 [81]. Specifically, residual Illumina-specific adaptors were clipped from the reads, leading and trailing bases with a Phred score less than 15 were removed and the read trimmed if a sliding window average Phred score over four bases was less than 20. Only reads where both pairs had a length greater than 36 bp post-filtering were retained. The recently assembled turbot genome [28] was used as a reference for read mapping. The genome sequence spanned 544 Mb and consisted of 16,493 scaffolds with N50 of 4.3 Mb (European Nucleotide Archive (ENA) project: PRJEB11743). Eighty percent of the genome assembly (156 scaffolds) was anchored to linkage groups (LG) of the turbot genetic map [27], enabling integrative and comparative mapping. Filtered reads were mapped to the genome using Tophat2 v2.0.12 [82] that leverages the short read aligner Bowtie2 v2.2.3 [83] with a maximum intron length of 20 kb. Cufflinks v2.2.1 [84] was used to build gene transfer format (GTF) files and cufflinks gffread utility was employed to obtain final Fasta files for muscle and liver transcriptomes.

### 3.3. Growth-Related Turbot Sequences

Growth-related genes were selected based on gene ontology (GO) criteria using Blast2GO [85], and previous reports on growth traits in fish [16,18,22,32] and their sequences retrieved from phylogenetically close model teleosts (stickleback, medaka, tetraodon) in Ensembl release 75 [86]. These sequences were used to scan both muscle and liver transcriptomes for orthologous turbot sequences using local BLAST (e-value < E^−10^) [77]. Once obtained, turbot growth-related sequences were blasted against the NCBI [87] non-redundant protein database to confirm their identity. Turbot gene sequences were also blasted against the turbot genome assembly to obtain their genomic position (unique significant hits with respect to specific genomic scaffolds).

### 3.4. Co-Localization of Candidate Genes with Growth QTLs

We checked for co-localization of the turbot growth-related genes in the genetic map with previously described growth QTL markers [29,30] also placed in the genome [28] using local BLAST (e-value < E^−10^) [88]. Genes which were placed in the same scaffold as growth QTL markers, preferably with a gene-marker distance below 1 Mbp, were considered as candidates to explain growth associations. Since no associated markers were reported for some growth QTLs (LG 6, 10, 14, 15, 22, 23), the closest markers to the estimated QTL position in the genetic map were used as references to predict gene co-localization. For those genes placed on genomic scaffolds not anchored to any turbot LG, comparative mapping against the most informative model fish for turbot (*Gasterosteus aculeatus* and *Oryzias latipes*) [26] was used to infer their syntenic position in the turbot genetic map (LG2, LG10, LG15, LG16, LG22). 

### 3.5. SNP Calling

SNP positions within the aligned reads were identified using the pileup function in SAMtools utilities v0.1.19 [89] with a Phredd base quality ≥20, sample read depth ≥10 and minor allele count ≥3. Reads from the two libraries (muscle and liver) were combined to increase coverage and confidence of SNP calling. SNPs located in turbot growth genes were inspected manually in the aligned reads using Tablet [90] to avoid genes with low minor allele frequencies, and their position within genes was investigated (UTR *vs.* coding regions) to select SNPs with higher chances of being biologically relevant.

### 3.6. SNP Genotyping and Validation

A total of 45 SNPs located in 45 different genes were selected based on previous specified criteria (functional relevance, co-localization with growth QTLs and SNP calling). DNA was extracted from a piece of caudal fin using standard phenol-chloroform procedures [91]. SNPs were validated and genotyped with the MassARRAY platform (Sequenom, San Diego, CA, USA) following the protocols and recommendations provided by the manufacturer. Briefly, the technique consists of an initial locus-specific polymerase chain reaction (PCR), followed by single-base extension using mass-modified dideoxynucleotide terminators of an oligonucleotide primer that anneals immediately upstream of the SNP [92,93]. The distinct mass of the extended primer identifies the SNP allele. MALDI-TOF mass spectrometry analysis in an Autoflex spectrometer was used for allele scoring. The 45 SNPs were combined in two multiplex reactions of 29 and 16 SNPs. SNPs were classified based on manual inspection as “failed assays” (in case that the majority of genotypes could not be scored and/or the samples did not cluster well according to genotype), and feasible SNPs (markers with proper and reliable genotypes), either monomorphic or polymorphic. All SNPs were genotyped in a population sample of 34 individuals from a wild Cantabrian population, different from the samples used for RNA-seq SNP detection (see above). The Cantabrian population had been previously used as reference for immune-related marker validation in turbot [38]. Genetic diversity (unbiased expected heterozygosity, He) and minimum allele frequency (MAF) were estimated using FSTAT 2.9.3 [94]. Departures from Hardy-Weinberg equilibrium (HW) were tested using GENEPOP 4.0 [95,96]. Linkage disequilibrium (association between genotypes at pairs of loci) was tested using the log-likelihood ratio G-statistic implemented in FSTAT 2.9.3. Conformance to HW was checked using the complete enumeration method implemented in GENEPOP 4.0, because all loci were biallelic. The fixation index FIS per locus was estimated using GENEPOP 4.0 and their significance using FSTAT 2.9.3. Bonferroni correction was applied when multiple tests were performed [97].

### 3.7. Data Accessibility

RNA sequencing project: ncbi BioSample accessions SAMN03740585 (muscle) and SAMN03740586 (liver). Sequenom assays for growth-related gene SNPs: Table S2, Supplementary Materials.

## 4. Conclusions

RNA-seq is an efficient technique to develop markers for candidate gene association studies related to targeted biological processes. A high number of SNPs were identified in the liver and muscle transcriptomes of turbot, which revealed enrichment in transcripts involved in growth regulation, and allowed for detection of genetic variation in several relevant growth-related genes of fish and vertebrate. Integrating genomic and linkage mapping resources allowed us to place the candidate genes in the turbot genetic map and to check for co-localization with growth QTLs in this species. SNP markers for 43 genes were validated in a turbot population, providing useful tools for fine mapping within QTL regions, population genomics studies, and functional and association analysis with growth phenotypes in turbot. 

## Figures and Tables

**Table 1:** RNA-seq and transcriptome assembly statistics.

Statistic	Muscle	Liver	Total
Raw reads	19,326,140	17,466,901	36,793,041
Trimmed reads	17,596,236 (91.0%)	16,398,082 (93.9%)	33,994,318 (92.4%)
Concordant aligned reads	15,570,568 (88.5%)	14,805,040 (90.3%)	30,375,608 (89.4%)
Number of genes	19,147	15,928	20,447
Number of transcripts	27,664	22,061	33,795
Minimum transcript size	62	78	62
Maximum transcript size	67,989	17,880	67,989
Transcripts over 500 bp	26,659	21,319	32,650
Mean transcript length (bp)	2594.66	2428.24	2819.60
N50	3411	3154	3691
N90	1381	1293	1531

Concordant aligned reads are pairs of reads which were assembled at the same genomic location and the percentage shown is calculated to the trimmed reads.

**Table 2:** Growth-related expressed genes selected for SNP screening in turbot.

Gene ^1^	Annotation	Organ ^2^	LG ^3^	QTL Marker ^4^	Distance ^5^ (kbp)	QTL Information ^6^ (VPE) ^†^
*acss3*	Acyl-CoA synthetase 3	M&L	16	SmaUSC-E11	909	BW-QTL (13%)
*actc*	Actin, alpha cardiac muscle 1	M	3	Sma-USC144	1995	K-QTL (12%)
*akirin2*	Akirin2	M&L	23	nd	nd	L-QTL interval
*akt3*	Ser/Thr-protein kinase	M&L	15	Sma-USC211	1621	BW, L-QTL (13%)
*atp2a1*	Calcium transporting ATPase1	M	21	Sma-USC234	4364	K-QTL interval
*chpt1*	Choline phosphotransferase 1	M&L	16 *	nd	nd	BW-QTL (13%)
*eno1*	Alpha-enolase	M&L	10	SmaUSC-E32	5651	K-QTL interval
*far2*	Fatty acyl CoA reductase 2	M&L	6	Sma-USC264	823	BW, L-QTL interval
*fbxo32*	F-box protein 32	M&L	2	Sma-USC242	1924	K-QTL interval
*fgf6b*	Fibrobalst growth factor 6b	M	16	SmaUSC-E11	34	K-QTL (25%)
*foxm1*	Forkhead box M1	M&L	16	SmaUSC-E11	762	BW-QTL (13%)
*foxo1b*	Forkhead Box O1b	M&L	8	Sma-USC194	1843	L-QTL interval
*ghr2*	Growth hormone receptor 2	M&L	14	nd	nd	BW, L-QTL interval
*got1a*	Glu-oxaloacetic transaminase 1a	M&L	2	Sma-USC112	622	BW, L-QTL interval
*igf1*	Insulin-like growth factor 1	L	16	SmaUSC-E11	1453	K-QTL (25%)
*igf2b*	Insulin-like growth factor 2b	M&L	6	nd	nd	BW, L-QTL interval
*igfbp2*	Igf binding protein 2	L	20	Sma-USC29	7275	L-QTL (8%)
*igfbp3*	Igf binding protein 3	M	1	Sma-USC15	413	BW-QTL (14%)
*insrb*	Insulin Receptor	M&L	5	Sma-USC247	2321	BW, L-QTL interval
*jak2a*	Janus kinase 2a	M&L	14	Sma-USC82	152	BW-QTL (11%)
*jak2b*	Janus kinase 2b	M&L	12	SmaUSC-E14	2037	L-QTL (13%)
*lepr*	Leptin receptor	M&L	5	Sma-USC65	488	BW, L-QTL interval
*lum*	Lumican	M&L	16	Sma-USC223	504	BW-QTL (13%)
*mef2a*	Myocite enhancer factor a	M&L	6	nd	nd	BW, L-QTL interval
*mir133b*	Mir133b	M	23	Sma-USC273	5066	BW, L-QTL (11%)
*mstn1*	Myostatin 1	M	20 *	nd	nd	L-QTL interval
*mtor*	Ser/Thr-protein kinase mTOR	M&L	10	nd	nd	F-QTL interval
*myod*	Myoblast determination protein	M	1	Sma-USC101	351	BW-QTL (8%)
*myog*	Myogenin	M	11	Sma-USC158	3209	K-QTL (10%)
*pah*	Phenylalanine hydroxylase	M&L	16	SmaUSC-E11	1511	BW-QTL (13%)
*pklr*	Pyruvate kinase	M&L	2	Sma-USC168	443	K-QTL interval
*pkm*	Pyruvate kinase a, muscle	M&L	4	Sma-USC7	2610	K-QTL (10%)
*ptn*	Pleiotrpophin.	L	16	SmaUSC-E11	693	BW-QTL (13%)
*pvalb1*	Parvalbumin 1	M	21	Sma-USC234	1911	K-QTL interval
*smad4b*	Mothers- decapentaplegic 4	M&L	14	Sma-USC63	589	BW-QTL (11%)
*sox5*	SRY-box 5	M&L	16	Sma-USC256	560	BW, L-QTL (8%)
*ssr3*	Translocon protein gamma	M&L	15 *	nd	nd	BW, L-QTL interal
*stat5.1*	Activator of transcription 5	M&L	21	Sma-USC41	125	K-QTL (4%)
*tgfb1*	Transforming growth factor β1	M&L	15	nd	nd	BW-QTL interval
*tgfb2*	Transforming growth factor β2	M&L	2 *	nd	nd	BW, L-QTL inteval
*tgfbr1*	Tgf β1 receptor	M&L	17	Sma-USC55	1569	L-QTL (9%)
*tgfbr2*	Tgf β2 receptor	M&L	22	nd	nd	Gene function ^†^
*tgfbr3*	Tgf β3 receptor	M&L	5	ScmM1	265	BW, L-QTL (11%)
*tnnc2*	Troponin C, skeletal muscle	M	10 *	nd	nd	K-QTL interval
*usp18*	Ubiquitin specific peptidase	M&L	16	SmaUSC-E11	84	BW-QTL (13%)

^1^ Gene symbol (Table S1, Supplementary Materials); ^2^ Organ expression: muscle (M) and/or liver (L); ^3^ Predicted Linkage Group (LG) containing the gene-specific scaffold (Table S1, Supplementary Materials) obtained either from anchor markers of the genetic map into the turbot genome or by comparative mapping against model fish (*); ^4^ Closest growth QTL marker within scaffold, if applicable (nd: Gene and QTL maker/s are not in the same scaffold); ^5^ Physical distance between genes and QTL markers; ^6^ Selection criteria: Gene function and/or distance to growth QTL (BW: Body weight, L: Length, K: Fulton’s factor [29,30]), either within QTL intervals or close to associated markers; VPE (%): Phenotypic variance explained by associated markers to growth traits, when applicable; ^†^ Outlier marker proposed to be under selection in turbot [32].

**Table 3:** SNP markers for growth-related genes in turbot.

SNP	Variant	Genomic Position ^1^	Gene Region ^2^	MAF ^3^	*p* (HW) ^4^	He ^5^	Fis ^6^
*actc*	T/C	Sm_46: 2,118,151	Exon syn	T = 0.368	1	0.471	−0.06
*akirin2*	C/G	Sm_26: 4,028,782	3’ UTR	G = 0.324	0.7061	0.445	0.074
*akt3*	C/T	Sm_31: 2,270,529	3’ UTR	T = 0.118	1	0.21	−0.119
*atp2a1*	A/T	Sm_11: 7,019,041	3’ UTR	A = 0.485	1	0.507	0.014
*chpt1*	G/A	Sm_183: 505,467	Exon syn	T = 0.338	0.2538	0.453	−0.234
*eno1*	C/G	Sm_4: 2,026,813	5’ UTR	C = 0.25	1	0.381	−0.005
*far2*	G/A	Sm_100: 790,917	Exon syn	A = 0.088	1	0.163	−0.082
*fgf6b*	G/A	Sm_49: 505,467	Exon R-Q	G = 1	-	-	-
*foxm1*	C/G	Sm_49: 1,237,830	5’ UTR	C = 0.344	0.1129	0.461	0.322
*foxo1b*	C/G	Sm_14: 4,371,288	Exon A-G	C = 0.353	1	0.463	−0.015
*ghr2*	G/A	Sm_67: 9082	Exon syn	G = 1	-	-	-
*got1a*	C/A	Sm_35: 2,520,843	3’ UTR	C = 1	-	-	-
*igf1*	G/T	Sm_49: 1,922,714	Intron	T = 0.029	1	0.058	−0.015
*igf2b*	G/A	Sm_15: 9,070,308	3’ UTR	A = 0.265	1	0.395	−0.043
*igfbp2*	A/G	Sm_2: 14,079,242	3’ UTR	G = 0.324	0.7061	0.445	0.074
*igfbp3*	C/A	Sm_1: 12,956,648	3’ UTR	A = 0.103	1	0.187	−0.1
*insrb*	G/A	Sm_5: 4,670,818	Exon syn	A = 0.132	1	0.233	−0.138
*jak2a*	C/G	Sm_38: 2,890,626	Intron	G = 0.441	0.7412	0.501	0.06
*jak2b*	A/T	Sm_18: 4,376,249	3’ UTR	A = 0.044	1	0.086	−0.031
*lepr*	A/G	Sm_5: 10,530,481	Exon V-A	A = G = 0.5	0.7387	0.508	0.074
*lum*	T/C	Sm_36: 3,958,348	5’ UTR	C = 0.029	1	0.058	−0.015
*mef2a*	C/G	Sm_15: 4,752,470	3’ UTR	G = 0.044	1	0.086	−0.031
*mir133b*	C/A	Sm_47: 179,652	5’ UTR	A = 0.059	1	0.112	−0.048
*mstn1*	A/T	Sm_275: 53,874	3’ UTR	A = 0.176	0.249	0.296	0.205
*mtor*	C/A	Sm_21: 2,840,205	Exon syn	A = 0.103	1	0.187	−0.1
*myod*	G/T	Sm_84: 1,867,326	Exon syn	G = 1	-	-	-
*myog*	A/T	Sm_9: 6,475,591	3’ UTR	T = 0.059	1	0.112	−0.048
*pah*	G/C	Sm_49: 1,912,258	5’ UTR	C = 0.162	1	0.275	0.039
*pklr*	C/T	Sm_6: 7,978,133	3’ UTR	C = 0.029	1	0.058	−0.015
*pkm*	A/C	Sm_32: 2,688,948	Intron	A = 0.439	0.7262	0.501	0.093
*ptn*	T/G	Sm_49: 1,163,341	3’ UTR	T = 0.25	0.0814	0.379	−0.32
*pvalb1*	C/T	Sm_11: 4,556,367	5’ UTR	T = 0.044	1	0.086	−0.031
*smad4b*	C/T	Sm_38: 1,262,602	5’ UTR	T = 0.074	1	0.138	−0.065
*sox5*	C/T	Sm_40: 1,284,941	Exon syn	C = 0.426	0.5019	0.496	−0.128
*ssr3*	C/T	Sm_55: 538,312	3’ UTR	T = 0.176	1	0.295	0.003
*stats5.1*	G/C	Sm_85: 414,378	3’ UTR	C = 0.309	0.686	0.434	0.119
*tgfb1*	A/C	Sm_28: 3,438,353	5’ UTR	C = 0.426	1	0.496	−0.007
*tgfb2*	A/C	Sm_58: 12,144	3’ UTR	C = 0.132	1	0.233	−0.138
*tgfbr1*	C/T	Sm_3: 2,957,935	3’ UTR	T = 0.029	1	0.058	−0.015
*tgfbr2*	T/A	Sm_10: 2,181,357	3’ UTR	T = 0.235	0.6469	0.365	−0.13
*tgfbr3*	T/C	Sm_34: 1,743,526	Intron	C = 0.338	0.7061	0.454	−0.102
*tnnc2*	T/C	Sm_174: 11,856	Exon syn	T = 0.368	1	0.471	−0.06
*usp18*	G/A	Sm_49: 555,569	Exon S-L	A = 0.191	0.3136	0.313	−0.222

^1^ SNP genome position (scaffold code: Position in pb); ^2^ SNP genic position (within exons synonymous mutations (syn) or amino acid substitutions are indicated); ^3^ Minimum allele frequency; ^4^ *p*-value for Hardy-Weinberg equilibrium (HW) test; ^5^ Unbiased genetic diversity; ^6^ Deviation from HW expected heterozygosity (Fixation index, F_IS_).

## Supplementary Materials

Supplementary materials can be found at http://www.mdpi.com/1422-0067/17/2/243/s1.
